# Hemophagocytic syndrome secondary to SARS-Cov-2 infection: a case report

**DOI:** 10.1186/s12879-021-06532-7

**Published:** 2021-08-13

**Authors:** Marco Meazza Prina, Francesca Martini, Federico Bracchi, Daniela Di Mauro, Anna Fargnoli, Marco Motta, Cristina Giussani, Giovanni Gobbin, Monica Taverna, Andrea D’Alessio

**Affiliations:** Department of Internal Medicine and Onco-Haematology Policlinico S.Marco, GSD University and Research Hospital, Corso Europa 7, 24040 Zingonia, BG Italy

**Keywords:** Hemophagocytic syndrome, COVID-19, Polyserositis, Case report

## Abstract

**Background:**

Hemophagocytic syndrome (HPS) is a severe hyperinflammatory disease, whose diagnosis is based on the HLH-2004 criteria. In secondary forms of HLH (sHLH), the primary goal is treating the triggering factors such as COVID-19 (Coronavirus disease 2019). The link between the cytokine storm related to COVID-19 and development of sHLH has already been reported since the onset of pandemic, but little is known about clinical manifestations of HLH which develop after the patient’s recovery from mild symptomatic or asymptomatic Sars-CoV-2 infection.

**Case presentation:**

We describe the case of a woman diagnosed with sHLH related to previous Sars-CoV-2 infection and successfully treated with steroids, colchicine, etoposide and ruxolitinib.

**Conclusions:**

Our report suggests that HLH-like syndrome might be secondary to Sars-CoV-2 infection, even if the patient utterly recovered from the mildly symptomatic viral infection. In addition, we underline the treatment with low dose ruxolitinib plus etoposide as a potential choice for Sars-CoV-2 infection related HLH.

## Background

Hemophagocytic syndrome (HPS) or hemophagocytic lymphohistiocytosis (HLH) is a severe hyperinflammatory disease, determined by a dysregulated activation of macrophages and cytotoxic T cells. Two kinds of HPS have been described: the primary form, caused by mutations affecting immune system and mainly common among paediatric patients and the secondary (or acquired) form, potentially triggered by several different diseases such as infections (mostly viral infections), malignancies (in particular malignant lymphomas), macrophage activation syndrome (MAS) in autoimmune/autoinflammatory disorders and novel immunotherapies (CART cells and blinatumumab) [1–6].

The diagnosis is based on the HLH-2004 criteria [7]. The most recent HLH probability score (*Hscore*) may be a helpful diagnostic tool at the patient's initial presentation [8]. Moreover, hyperbilirubinemia, hepatomegaly, elevated LDH and D-dimer levels are common features of HLH, even if not included in HLH-2004 criteria. Mortality of HLH remains high in adults, around 70%, despite therapy [9]. In secondary forms of HLH (sHLH), the primary goal is treating the triggering factors such as Sars-CoV-2 infection. The link between the cytokine storm related to COVID-19 and development of sHLH has already been reported since the onset of pandemic [9], but little is known about clinical manifestations of HLH which develop after patient’s recovery from Sars-CoV-2 infection. Of note, in COVID-19 related sHLH, hemophagocytosis on bone marrow biopsy has not been reported so far [10]. Early high dose steroids and IVIG (1 g/kg) for 2 days are considered the first line treatment of sHLH. The first cases of macrophage-activation syndrome (MAS) in association with COVID-19 were treated successfully with JAK inhibitors and IL-1 or IL-6 blockers [11, 12].

Low dose ruxolitinib plus HLH-94 protocol has already been reported as a potential choice for sHLH [13]. Finally, in patients with severe active disease, a reduced dose of Etoposide (50–100 mg/mq once weekly) may be very effective [14].

## Case presentation

### Patient information

In May 2020 a 56-year-old Caucasian female without any relevant past medical history (no drugs taken or previous diseases referred), was hospitalized for fever up to 40 °C, dry cough, ageusia and anosmia at Policlinic San Marco Hospital, Zingonia (Bergamo, Italy). No family history of diabetes, cardiovascular diseases, cancer, autoimmune disease (such as arthritis, vasculitis or lupus) or other relevant diseases were reported. She reached menopause at about 52 years old; her previous pregnancies proceeded without any complications. She has never smoked and she drinks alcoholic drinks occasionally (a glass of wine twice a week).

### Clinical findings

The patient’s clinical picture was characterized by fever up to 40 °C, dry cough, ageusia and anosmia, which were significative of COVID-19.

### Diagnostic assessment

During the hospitalization, total body CT-scan revealed polyserositis: pericardial effusion, pleuro-parenchymal fibrosis of the lung bases associated with bilateral pleural effusion and splenomegaly. Three SARS-Cov-2 nasopharyngeal swabs were performed and each of them resulted negative and multiple blood culture sets resulted negative. Viral infections (Epstein Barr virus EBV, Cytomegalovirus CMV, Human Immunodeficiency virus HIV, hepatotropic viruses and viruses transmitted by arthropods) were promptly ruled out, as well as atypical pneumonia by Mycoplasma or Chlamydia bacteria. Considering that Bergamo and nearby cities have been the most affected area in Italy by COVID-19, SARS-Cov-2 immunoglobulins G (IgG) were tested and found to be positive. In the meantime, we observed lowering fibrinogen values, occurrence of cytopenias (anaemia < 10 g/dL and thrombocytopenia < 100.000/mcl), increasing values of ferritin aspartate aminotransferase and triglycerides. Moreover, LDH and D-dimer were elevated, direct and indirect Coombs tests were negative, no schystocytes were observed at the morphological exam of the patient’s blood smear, aptoglobin, bilirubin and reticulocytes were normal, activated partial thromboplastin time was slightly elevated (according to the inflammatory state) while prothrombin time was normal. Antinucleus antibodies, rheumatic factor, lupus anticoagulant and anti-cardiolipin antibodies were found negative. The individual risk of HLH the patient scored 269 points with > 99% probability of having the syndrome (*HScore* see Tables 1 and 2). *HScore* greater than 169 is 93% sensitive and 86% specific for hemophagocytic syndrome. Therefore, a bone marrow biopsy was performed: the morphological and histological exams did not revealed aspects of hemophagocytosis. Functional (NK cell cytotoxicity and CD107a upregulation) and genetic testing were not performed, considering that abnormalities are rarely detected in adult patients and these tests are not generally recommended in adult HLH [14]. Moreover, in this particular historical period of global health crisis the execution of more advanced tests such as next generation sequencing was not promptly available. A diagnosis of HLH was made according to HLH-2004 criteria (presence of fever, splenomegaly, cytopenias, hypofibrinogenemia, hypertriglyceridemia, ferritin > 500 mcg/L) and HScore.

**Table. 1:** HScore for reactive hemophagocytic syndrome

Variable	Points		Patient’s score
Known underlying immunosuppression HIV positive or receiving long‐term immunosuppressive therapy (i.e., glucocorticoids, cyclosporine, azathioprine)	No	0	No
	Yes	+ 18	
Temperature, °F (°C)	< 101.1 (< 38.4)	0	
	101.1–102.9 (38.4–39.4)	+ 33	
	> 102.9 (> 39.4)	+ 49	39.5 °C
Organomegaly	No	0	
	Hepatomegaly or splenomegaly	+ 23	Splenomegaly
	Hepatomegaly and splenomegaly	+ 38	
Number of cytopenias Defined as hemoglobin ≤ 9.2 g/dL (≤ 5.71 mmol/L) and/or WBC ≤ 5000/mm^3^ and/or platelets ≤ 110,000/mm^3^	1 lineage	0	
	2 lineages	+ 24	
	3 lineages	+ 34	Hb 7.7 g/dL, WBC 3700/mm^3^, platelets 27,000/mm^3^
Ferritin, ng/mL (or μg/L)	< 2,000	0	
	2000–6000	+ 35	
	> 6000	+ 50	20,696 ng/mL
Triglyceride, mg/dL (mmol/L)	< 132.7 (< 1.5)	0	
	132.7–354 (1.5–4)	+ 44	
	> 354 (> 4)	+ 64	428 mg/dL
Fibrinogen, mg/dL (g/L)	> 250 (> 2.5)	0	
	≤ 250 (≤ 2.5)	+ 30	74 mg/dL
AST (aspartate aminotransferase), U/L	< 30	0	
	≥ 30	+ 19	59 U/L
Hemophagocytosis features on bone marrow aspirate	No	0	No
	Yes	+ 35	
Total			269 points (> 99% probability of hemophagocytic syndrome)

**Table 2:** HScore interpretation

Hscore	Probability of hemophagocytic syndrome (%)
≤ 90	< 1
91–100	~ 1
101–110	1–3
111–120	3–5
121–130	5–9
131–140	9–16
141–150	16–25
151–160	25–40
161–170	40–54
171–180	54–70
181–190	70–80
191–200	80–88
201–210	88–93
211–220	93–96
221–230	96–98
231–240	98–99
≥ 241	> 99

### Therapeutic interventions

**Fig. 1:**
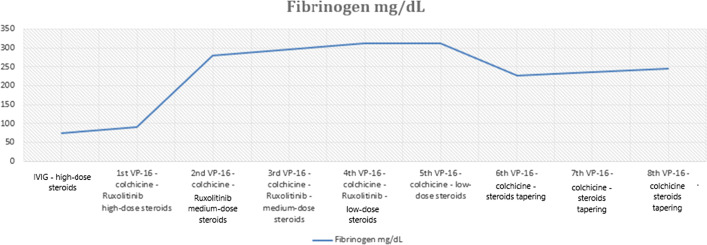
Patient’s laboratory Fibrinogen response to treatment with: IVIG; VP-16 100 mg/mq once a week; high-dose steroids: 10 mg/mq; medium-dose steroids: 5 mg/mq once a day; low-dose steroids: 2.5 mg/mq once a day; Ruxolitinib: 5 mg BID (followed by dose tapering 5 mg/die over 10 days); Colchicine: 1 mg once a day. Fibrinogen normal range: 180–350 mg/dL

**Fig. 2:**
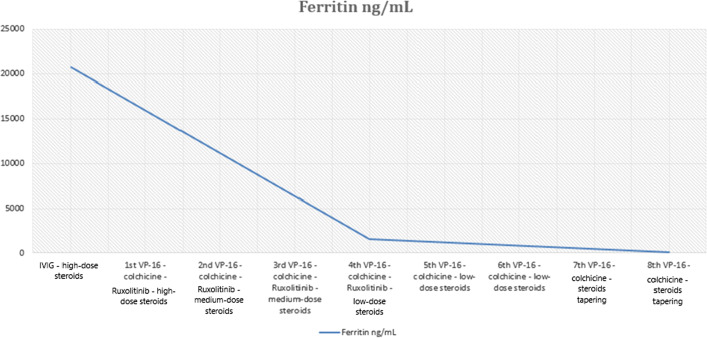
Patient’s laboratory ferritin response to treatment with: IVIG; VP-16 100 mg/mq once a week; high-dose steroids: 10 mg/mq; medium-dose steroids: 5 mg/mq once a day; low-dose steroids: 2.5 mg/mq once a day; Ruxolitinib: 5 mg BID (followed by dose tapering 5 mg/die over 10 days); Colchicine: 1 mg once a day. Ferritin normal range: 7–130 mg/dL

At the beginning the patient was treated with empiric antibiotic therapy (ceftriaxone), without any improvement of her clinical conditions and further empiric antibiotic regimens (piperacillin-tazobactam, teicoplanin, meropenem, linezolid, levofloxacin) were unsuccessful. The patient was diagnosed with hemophagocytic syndrome likely related to SARS-Cov-2 infection as a trigger factor and was treated with high-dose steroids (dexamethasone 10 mg/mq once a day D1–14, 5 mg/mq D15–28, 2.5 mg/mq after 1 month for maintenance treatment followed by slow reduction) and high dose intravenous immunoglobulins (IVIG) 1 g/kg for 2 consecutive days. Due to the persistence of fever and a further drop in platelets haemoglobin and fibrinogen after a week from the beginning of the immunosuppressive therapy, low-dose Ruxolitinib (5 mg bid) for a month, which was discontinued after a tapering over 10 days (5 mg/die), and Etoposide (VP-16) 100 mg/mq once weekly for 8 weeks were added to the treatment, while colchicine (1 mg once a day) therapy was administered for 3 months, considering the reported efficacy in the treatment of acute pericarditis [15]. In addition, antiviral (acyclovir 400 mg PO BID), antibiotic (sulfamethoxazole/trimethoprim three times weekly) and heparin (enoxaparin 4000 UI sc/die) prophylaxes were prescribed. During the treatment a remarkable improvement in the patient’s symptoms as well as the normalization of blood count and of fibrinogen and ferritin values (see Figs. 1 and 2), the disappearance of fever and the remission of polyserositis.

### Follow-up and outcomes

After the end of the treatment, the patient has been followed on a regular basis and the disease is still in remission. Serial blood tests revealed normal whole blood count (WBC) and normal values of fibrinogen and ferritin (see Figs. 1 and 2). In addition, CT scans at 3 and 6 months after the end of treatment as well as the echocardiography documented the remission of the patient’s polyserositis and of splenomegaly.

## Discussion and conclusions

The diagnosis of HLH can be very challenging. An increased awareness of this disease together with a rapid therapeutic approach can improve the prognosis [9]. Nevertheless, HLH is still a severe and potentially fatal disease. A diagnosis of HLH was made according to HLH-2004 criteria (presence of fever, splenomegaly, cytopenias, hypofibrinogenemia, hypertriglyceridemia, ferritin > 500 mcg/L) and HScore. Thrombotic thrombocytopenic purpura, disseminated intravascular coagulation, autoimmune haemolytic anemia and thrombocytopenia (Evans’ syndrome) as well as catastrophic antiphospholipid syndrome were considered as part of differential diagnosis. Even linezolid myelosuppressive side effects were valuated as a cause of cytopenia and therefore the drug was promptly suspended when it was shown inefficacious for the treatment of the patient’s fever. Since other common causes of sHLH (malignancies, viral infections or autoimmune-rheumatologic diseases) were excluded, we suggest that HLH-like syndrome could develop at a distance from the Sars-Cov-2 infection, as a result of persistent inflammatory state. In addition, we underline the treatment with low dose ruxolitinib plus etoposide as a potential choice for COVID-19 related HLH [13, 14]. Noteworthy, it is important to collect more information about potential persistent inflammatory state related to previous COVID-19 and therefore develop and improve therapy of HLH secondary to Sars-Cov-2 infection.

## Data Availability

The datasets used and/or analysed during the current study are available from the corresponding author on reasonable request.

## Abbreviations

HPS: Hemophagocytic syndrome
sHLH: Secondary forms of HLH
COVID-19: Coronavirus disease 19
MAS: Macrophage-activation syndrome
CT: Computed tomography
SARS: Severe acute respiratory syndrome
EBV: Epstein Barr virus
CMV: Cytomegalovirus
HIV: Human immunodeficiency virus
IVIG: Intravenous immunoglobulins
